# Biopsychological correlates of repetitive and restricted behaviors in autism spectrum disorders

**DOI:** 10.1002/brb3.2341

**Published:** 2021-09-02

**Authors:** Miguel Comparan‐Meza, Ivette Vargas de la Cruz, Fernando Jauregui‐Huerta, Rocio E. Gonzalez‐Castañeda, Oscar Gonzalez‐Perez, Alma Y. Galvez‐Contreras

**Affiliations:** ^1^ Maestría en Neuropsicología Departamento de Neurociencias, Centro Universitario de Ciencias de la Salud (CUCS) Universidad de Guadalajara Guadalajara JAL Mexico; ^2^ Unidad de Atención en Neurociencias Departamento de Neurociencias, Centro Universitario de Ciencias de la Salud (CUCS) Universidad de Guadalajara Guadalajara JAL Mexico; ^3^ Laboratorio de Microscopia de Alta Resolución Departamento de Neurociencias, Centro Universitario de Ciencias de la Salud (CUCS) Universidad de Guadalajara Guadalajara JAL Mexico; ^4^ Laboratorio de Neurociencias Facultad de Psicología Universidad de Colima Colima COL Mexico

**Keywords:** autism spectrum disorder, neurodevelopmental disorders, repetitive behaviors, restricted behaviors, stereotypes

## Abstract

**Background:**

Autism Spectrum Disorder (ASD) is considered a neurodevelopmental condition that is characterized by alterations in social interaction and communication, as well as patterns of restrictive and repetitive behaviors (RRBs). RRBs are defined as broad behaviors that comprise stereotypies, insistence on sameness, and attachment to objects or routines. RRBs can be divided into lower‐level behaviors (motor, sensory, and object‐manipulation behaviors) and higher‐level behaviors (restrictive interests, insistence on sameness, and repetitive language). According to the DSM‐5, the grade of severity in ASD partially depends on the frequency of RRBs and their consequences for disrupting the life of patients, affecting their adaptive skills, and increasing the need for parental support.

**Methods:**

We conducted a systematic review to examine the biopsychological correlates of the symptomatic domains of RRBs according to the type of RRBs (lower‐ or higher‐level). We searched for articles from the National Library of Medicine (PubMed) using the terms: autism spectrum disorders, ASD, and autism‐related to executive functions, inhibitory control, inflexibility, cognitive flexibility, hyper or hypo connectivity, and behavioral approaches. For describing the pathophysiological mechanism of ASD, we also included animal models and followed PRISMA guidelines.

**Results:**

One hundred and thirty‐one articles were analyzed to explain the etiology, continuance, and clinical evolution of these behaviors observed in ASD patients throughout life.

**Conclusions:**

Biopsychological correlates involved in the origin of RRBs include alterations in a) neurotransmission system, b) brain volume, c) inadequate levels of growth factors, d) hypo‐ or hyper‐neural connectivity, e) impairments in behavioral inhibition, cognitive flexibility, and monitoring and f) non‐stimulating environments. Understanding these lower‐ and higher‐level of RRBs can help professionals to improve or design novel therapeutic strategies.

## Graphical abstract

The psychobiological mechanisms that underly lower‐ or higher‐level behaviors in autism spectrum disorders (ASD) are not completely understood. Herein, the neurobiological, neuropsychological, and behavioral evidence that sustains the etiology of these behaviors in the pathogenesis of ASD are summarized.

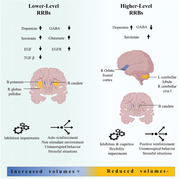

## 1 INTRODUCTION

Autism spectrum disorders (ASD) is defined by the Diagnostics and Statistics Manual of Psychiatric Disorders in their fifth edition (DSM‐5) as a group of neurodevelopmental disorders characterized by deficits in social communication and interactions as well as restricted and repetitive behaviors (RRBs) (American Psychiatric Association, 2013). The term ASD includes autistic disorder, Asperger's disorder, pervasive developmental disorder—not otherwise specified (PDD‐NOS), Rett´s disorder, and the childhood disintegrative disorder, which were considered subtypes of developmental disorders in the previous version (DSM‐IV‐TR). Early diagnosis of RRBs in autism is important, as these symptoms are considered predictive of anxiety in children (Baribeau et al., 2020) and adults (Kuzminskaite et al., 2020). To date, RRBs are considered some of the most disabling symptoms that affect patients’ relatives, financial stability, romantic relationships, and leisure activities (Bishop et al., 2007). In consequence, RRBs impair severely the mental health and clinical status of ASD patients.

**FIGURE 1:**
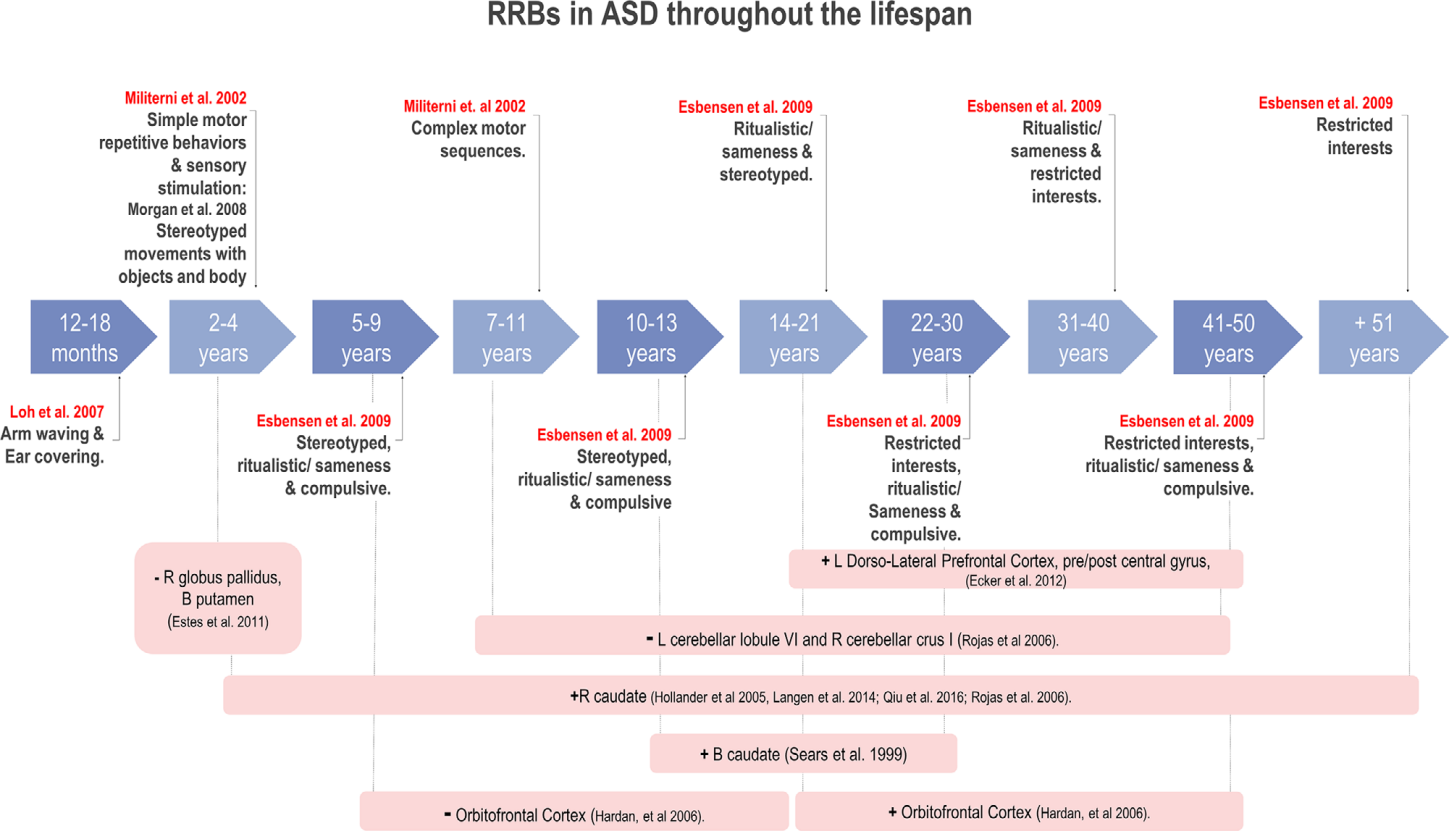
Timeline showing the type of RRBs found in ASD and the brain regions affected according to age. Dotted lines represent the increased (**+**) or decreased (−) volume of cortical and subcortical structures by age group. B, bilateral; L, left; R, right

In 1999, Turner proposed that RRBs involve a wide range of behaviors that are characterized by repetition, cognitive inflexibility, and social inappropriateness and can be classified into *lower‐level behaviors* that involve stereotypes and repetitive use of objects (stereotypical movements, self‐injurious behaviors, tics, repetitive object manipulation, and spontaneous dyskinesias), and *higher‐level behaviors* which refer to adherence to rituals or routines and excessive concern for a topic or an object (attachment to a specific object, insistence on the maintenance of sameness, repetitive language, and circumscribed interests) (Jiujias et al., 2017; Turner, 1999). The type of RRBs that are manifested in ASD depends on the developmental stage and gender. In this sense, 12‐month‐old toddlers (who later met diagnostic criteria for ASD) manifest an increase in arm waving and, at the 18th month, they present more significant ear covering behaviors (Loh et al., 2007). From the 12th to 18th month, most of these patients also showed more repetitive stereotyped movements (Morgan et al., 2008). At 4 years, ASD patients displayed more variety of simple motor‐repetitive and sensory‐stimulant behaviors. From the age of 5 to 9 years, patients start showing lower‐level RRBs whereas, in late childhood (<11 years old), they show lower‐ and higher‐level RRBs, such as ritualistic/sameness and compulsive behaviors, filling and emptying sequences, collecting and dispersing things, and building blocks or puzzles (Militerni et al., 2002). At the adult stage, people with ASD show a decrease in the frequency of lower‐level manifestations and more higher‐level RRBs (Esbensen et al., 2009) (Figure 1). Curiously, compulsive and self‐injurious behaviors remain stable throughout life (Lam & Aman, 2007). Finally, RRBs may affect socialization and daily living skills in children (Hong & Matson, 2021) and adolescents (Siracusano et al., 2021), but clinical or educational interventions help reduce their severity (Park et al., 2020).

According to the patients’ gender, emerging evidence suggests there exists differences in the RRBs pattern in later development stages. Specifically, boys above 5 years old show more frequency in RRBs as compared to girls, whereas 6‐year‐old girls show less severity in RRBs (Van Wijngaarden‐Cremers et al., 2014). This evidence suggests that the RRBs are less frequent or severe from the sixth year of life in women. According to the type of RRBs, male patients commonly displayed toy lining (Mandy et al., 2012), hand and finger movements, and fascination with movement or object usage (lower‐level RRBs) (Antezana et al., 2019), which are typically accompanied by higher‐level RRBs, such as storing a large amount of factual information (Mandy et al., 2012) and fascination with one subject/activity (Antezana et al., 2019). In contrast, female patients show more self‐injuring behaviors (hair and skin pulling), a type of lower‐level RRB, and some higher‐level RRBs such as compulsive (hoarding and saving) and insistence on sameness (strongly attached to one object or sitting in the same place). Some authors propose that these gender differences in RRBs could be associated with a delay in the diagnosis in women patients (Mandy et al., 2012). In summary, these differential patterns along the life span of RRBs seem to be associated with impairments and changes in the structure and function of the brain. Hence, RRBs are one of the two main symptomatic domains that define ASD (American Psychiatric Association, 2013) and the number, frequency, and severity of these behavioral manifestations determine the degree of independence and support that ASD patients will require. This review aims to compile the biopsychological correlates that may help explain the origin, type, maintenance, and clinical evolution of RRBs throughout the lifespan of ASD patients. Early detection of RRBs is a crucial step to adjust the therapeutical approach and reduce the magnitude of new comorbid symptoms in ASD patients.

## 2 METHOD

For this systematic review, we selected specialized literature from the National Library of Medicine (PubMed). The search was made using the terms: autism spectrum disorders, ASD, and autism‐related to executive functions, inhibitory control, inflexibility, cognitive flexibility, hyper‐ or hypoconnectivity, and behavioral approaches. The selection criteria for the articles were the relationship to the symptomatic domains of RRBs, the type of RRBs (lower or higher level), and the severity of symptoms. We also subcategorized these studies according to age, that is, childhood, adolescence, adulthood. For describing the pathophysiological mechanism of ASD, we also included animal models. The keywords were combined using the “OR” and “AND” commands in the database. The selection of articles was limited to the English language and studies that only explained deficits in communication or social interaction were excluded.

## 3 ETIOLOGY OF RRBS IN ASD

RRBs are commonly observed in other psychiatric disorders such as obsessive‐compulsive disorder (OCD), Gilles de la Tourette syndrome (GTS), and attention‐deficit hyperactivity disorder (ADHD). Contrasting to OCD, repetitive and ritualistic behaviors perceived in ASD are mainly associated with social deficits (Kim et al., 2016) and associated slightly with sophisticated compulsions and obsessions (Zandt et al., 2007). In both OCD and ASD, the RRBs become more complex as individuals get older, but in ASD the RRBs are regulated by the level of intelligence quotient (IQ). Conversely, the obsessions in OCD patients remain problematical regardless of age, while compulsions are more manageable throughout neurological development. Interestingly, when ASD and OCD are comorbid, these patients present more higher‐level RRBs throughout development (Jiujias et al., 2017), which suggests that the underlying mechanism of RRBs may indicate some discrete biopsychological differences between these disorders. On the other hand, GTS is characterized by motor and vocal tics and this disorder is comorbid with ASD or OCD. A recent study found that patients with GTS plus OCD show similar scores in unusual interest, repetitive stereotyped behavior, and compulsions as compared to ASD patients. This evidence suggests that the combination of GTS + OCD may share some biological mechanisms with ASD (Gulisano et al., 2020). ASD patients also show hyperactivity and inattention, two core symptoms of ADHD, and 30%–80% of ASD patients present ADHD as a comorbid disorder, which may indicate that some etiological processes underlie these disorders (Tsai et al., 2020).

At the biological level, alterations in several neurotransmission systems are linked with the excitatory/inhibitory imbalance hypothesis of ASD (Rubenstein, 2010; Rubenstein & Merzenich, 2003). Impairments in the dopamine system are related to RRBs. Dopamine modulates connectivity of several brain areas such as the ventral tegmental area, substantia nigra, striatum, nucleus accumbens, caudate, anterior cingulate cortex, dorsal prefrontal cortex (DLPC), globus pallidus, orbitofrontal cortex (OFC), putamen, subthalamic nucleus, thalamus, amygdala, and hippocampus (Haber, 2014). These dopaminergic circuits modulate the motor process (Wüllner et al., 1994), and alterations in dopamine projections from the substantia nigra pars compacta to striatum have been associated with RRBs (Pavǎl, 2017). Serotonin (5‐HT) is a neurotransmitter that also modifies the neural activity in the substantia nigra, hypothalamus, thalamus, hippocampus, amygdala, (Lidov & Molliver, 1982; Steinbusch, 1981), globus pallidus, caudate nucleus, putamen (Mori et al., 1985), and frontoparietal regions (Descarries et al., 1975), which modulate the welfare state (Marcet Rius et al., 2018). These alterations in the serotoninergic system have been associated with ASD (Page et al., 2009). Specifically, children, adolescents, and early adult patients present high levels of serotonin (Ciaranello, 1982; Ritvo et al., 1970; Schain & Freedman, 1961), which are inversely related to RRBs (Kolevzon et al., 2010).

Evidence obtained in toddlerhood, childhood, and adolescence has indicated an abnormal expression of GABA, the main inhibitory neurotransmitter in the brain (Krnjević & Schwartz, 1967). Low levels of GABA in the frontal lobe (Harada et al., 2011) and the first transverse temporal gyrus (an auditory cortex) have been reported in children with ASD (Rojas et al., 2014). These alterations may be associated with an impairment in GABA receptors. Children and adults with ASD show a disequilibrium in the expression of the GABRB3 subunit (Cook et al., 1998). Interestingly, the severity in RRBs correlates with high expression levels of GABRB3 (Shao et al., 2003), whereas in animal models for ASD, a reduction (30%–40%) in the release of GABA into the striatum produces RRB‐like behaviors (Chao et al., 2010). Glutamate is the main excitatory neurotransmitter in the brain (Curtis & Watkins, 1960). High levels of glutamate have been reported in children (Moreno‐Fuenmayor et al., 1996) and adult patients with ASD (Shinohe et al., 2006). In mice, the reduced expression of glutamate transporter 1 (GLT1) in astrocytes of the somatosensorial cortex, striatum, and thalamus increases the glutamatergic activity in neurons and produces behavioral changes that include a higher frequency of RRBs (Aida et al., 2015), suggesting that hyper glutamatergic activity via NMDA is associated with RRBs. These changes in neurotransmission and their relation to RRBs support the hypothesis that the imbalance in the inhibition and excitation levels of several neurotransmitters are involved in ASD.

**FIGURE 2:**
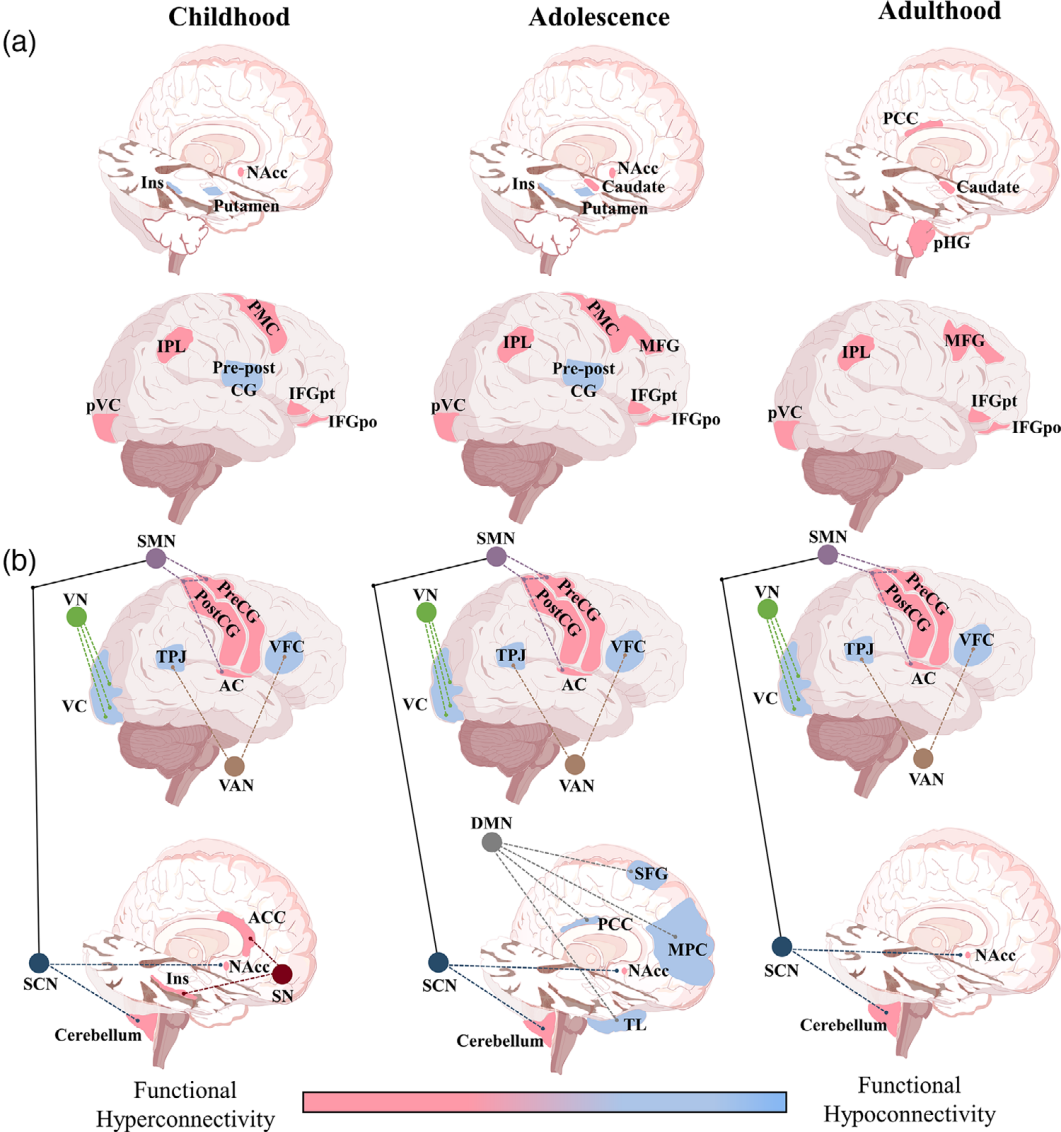
Brain schemes showing patterns of functional hypo‐ and hyperconnectivity between cortical and subcortical regions associated with RRBs at different developmental stages in patients with ASD. (a) In childhood and adolescence, hypoconnectivity among the precentral gyrus, postcentral gyrus, left posterior insula, and left posterior putamen is associated with stereotypies (a lower‐level RRB) and compulsive‐restrictive behaviors (higher‐level RRBs), whereas hypoconnectivity among the left precentral gyrus, left postcentral gyrus, left posterior insula, and left posterior putamen is associated with ritualistic behaviors (higher‐level RRBs). Otherwise, hyperconnectivity between the left NAcc and right PMC is associated with not‐specified RBBs. In childhood, adolescence, and adulthood, hyperconnectivity between the left pVC and right IFGpo is associated with not‐specified RRBs, whereas hyperconnectivity between the right IPL and right IFGpt is related to higher‐level RRBs. In adolescence and adulthood, hyperconnectivity between the right MFG and right caudate nucleus is associated with unstipulated RRBs. In adulthood, hyperconnectivity between the PCC and right pHG is associated with not‐specified RRBs. (b) Patterns of hypo‐ and hyperconnectivity intra and/or inter functional networks. In children, hyperconnectivity in the SN is associated with no specified RRBs, whereas, in adolescents, hypoconnectivity in the DMN is associated with undetermined RRBs. In childhood, adolescence, and adulthood, hypoconnectivity in the VN and in the VAN, and hyperconnectivity between the SCN and SMN are associated with ritualism, maintenance of sameness, repetitive language, circumscribed interests, and compulsions (higher‐levels RRBs) and stereotypies, mannerism, and self‐injurious behavior (lower‐level RRBs) (Abbott et al., 2018; Akkermans et al., 2019; Delmonte et al., 2013; Monk et al., 2009; S. Tang et al., 2020; Traynor et al., 2018; Uddin et al., 2013; Weng et al., 2010). Blue color represents hypoconnectivity and red color indicates hyperconnectivity. Violet circles and dotted lines represent the SMN. Green circles and dotted lines represent the VN. Brown circles and dotted lines in brown represent the VAN. Gray circle and dotted lines represent the DMN. Dark red circle and dotted lines represent the SN. Dark blue circle and dotted lines represent the SCN. Black solid lines indicate connectivity among cerebral networks. **Brain regions**: AC, auditory cortex; ACC, anterior cingulate cortex; IFGpo, inferior frontal gyrus pars orbitalis; IFGpt, inferior frontal gyrus pars triangularis; Ins, insula; IPL, inferior parietal lobule; MFG, middle frontal gyrus; MPC, medial prefrontal cortex; NAcc, nucleus accumbens; PCC, posterior cingulate cortex; pHG, parahippocampal gyrus; PMC, premotor cortex; pVC, primary visual cortex; Pre‐Post CG, pre and post central gyrus; SFG, superior frontal gyrus; TL, temporal lobe; TPJ, temporoparietal junction; VC, visual cortices; VFC, ventral frontal cortex. **Brain networks**: DMN, default mode network; SCN, subcortical network; SMN, somatomotor network; SN, salient network; VAN, ventral attention network; VN, visual network

A fundamental finding in ASD is the abnormal size and cellular differentiation in several brain regions (Fang et al., 2014). The most common anomalies include a large head circumference (Courchesne et al., 2001) and overgrowth of frontal, temporal, parietal, and occipital lobes (Carper et al., 2002). In ASD patients, abnormal gray matter volumes have been related to the type of RRBs (Hardan et al., 2006) or their severity (Estes et al., 2011). Remarkably, abnormalities in the brain size have been proposed as the origin of alterations in brain connectivity (Abbott et al., 2018; Akkermans et al., 2019; Delmonte et al., 2013; Monk et al., 2009; S. Tang et al., 2020; Traynor et al., 2018; Uddin et al., 2013; Weng et al., 2010). The connectivity hypothesis in ASD suggests the existence of abnormal patterns of connectivity that, in turn, can produce the core symptoms of this pathology (Horwitz et al., 1988; Just et al., 2004). Recent findings indicate that a decrease in microstructural integrity among neural connections of the dorsal premotor and the supplementary motor area with the corticospinal tract correlates positively with the severity of RRBs showed by adult ASD patients (Hau et al., 2021). Evidence of hypo‐ or hyperconnectivity related to the type of RRBs in ASD has been schematized in Figure 2.

At the neuropsychological level, one of the hypotheses that explain RRBs in ASD is the dysfunction in executive functions (Ozonoff et al., 1991). Executive functions comprise impulse control, inhibition of prepotent (but irrelevant responses), flexibility of thoughts, and behavior (Ozonoff et al., 1991). These skills help people to set up, make, and execute plans to elicit purposive and self‐directed behaviors (Lezak, 1982). The deficit in inhibition has been associated with the inefficient ability to regulate RRBs (Turner, 1997). This hypothesis was supported by Valeri and coworkers, who found that children with ASD committed more errors to suppress responses in comparison to typically developed children (Valeri et al., 2019). Similar findings have been documented in adults (Van Eylen et al., 2015). Cognitive flexibility alludes to the capacity of an individual for switching between behaviors, thoughts, or tasks according to the new dynamic environmental needs (Monsell, 1996, 2003). Patients with ASD show rigid behavior due to cognitive flexibility impairments, which could explain their problems for plan changing in daily activities (Geurts et al., 2009). Children with ASD present poor cognitive flexibility that is associated with problems for integrating complex rules and developing proper responses (Faja & Dawson, 2014). At this developmental stage, they present problems for switching recompenses from positive to negative values that, in turn, are related to the severity of RRBs (Yerys et al., 2009). Interestingly, cognitive inflexibility persists in adults with ASD (Fujino et al., 2019), which supports the notion that ASD patients display strong intellectual rigidity across the lifespan.

Monitoring response is the ability to examine the effects of an action and validate its compatibility with their intentions (Botvinicket al., 2001). During childhood and adolescence, subjects with ASD show deficient action monitoring, and consequently they commit more errors and tend to improperly correct them as compared to typically developed subjects (Russell & Jarrold, 1998). These findings suggest that subjects with ASD ignore the consequences of their actions and do not tend to not correct or adjust their actions to improve their performance (Russell & Jarrold, 1998). Hence, this evidence supports the notion that inhibition, cognitive flexibility, and monitoring response problems are neuropsychological features of RRBs that promote the overlearning of routines or improper behaviors in people with ASD throughout their lifespan. Abnormal cognitive inhibition and flexibility related to RRBs have been corroborated in a recent meta‐analysis, which found that high scores in the scale of RRBs in ASD patients correlated to high scores in their parental report of two cognitive characteristics, set‐shifting, and inhibitory control (Iversen & Lewis, 2021).

At the behavioral level and from the perspective of functional analysis it has been suggested that RRBs are self‐shaped and self‐maintained via auto‐stimulation and perceptual reinforcement (Lovaas et al., 1987). This hypothesis proposes that RRBs can be maintained by the exposure to stimuli involved in a novel situation, which enhances responses to stress or anxiety (Prizant, 1996) and are more frequent in non‐stimulating environments (Horner, 1980).

## 4 BIOPSYCHOLOGICAL CORRELATES OF LOWER‐LEVEL BEHAVIORS

As we mentioned above, one of the main hypotheses for explaining RRBs consist of alterations in several neurotransmission systems related to the excitatory/inhibitory imbalance (Rubenstein, 2010; Rubenstein & Merzenich, 2003). Alterations in dopamine projections have been associated with lower‐level RRBs (Pavǎl, 2017). Intrastriatal administration of a dopamine receptor D1 (DRD1) antagonist decreases spontaneous stereotypies without affecting non‐stereotypic motor behavior (Presti et al., 2003). In contrast, ASD patients treated with risperidone, an antagonist of the dopamine receptor D2 (DRD2), show a decrease in sensory–motor behaviors such as hand flapping, rocking, pacing, rubbing, and sniffing surfaces (McDougle et al., 2005), and self‐injurious behaviors (Canitano, 2006). Children with ASD also show an alteration in D4 receptors (DRD4) that are associated with an increase in the frequency of tics (Gadow et al., 2010). A combination of polymorphisms for DRD4 and the protein transporter for dopamine (DAT1) has been associated with more severe tics as compared to those individuals with only one polymorphism (Gadow et al., 2010). This evidence suggests that the co‐existence of polymorphism in the dopaminergic system seems to be associated with the severity of this type of RRBs.

On the other hand, levels of serotonin in ASD patients are inversely related to self‐injurious behaviors, which suggests that this neurotransmitter or the neural circuits that regulate serotoninergic neurons are involved in these behaviors (Kolevzon et al., 2010). In this regard, a reduction in the release of GABA into the striatum has been related to self‐injurious, stereotypies, and compulsive behaviors in mice (Chao et al., 2010). In animal models, a reduction in the expression of GLT1 in astrocytes of the somatosensorial cortex, striatum, and thalamus increases the glutamatergic activity in neurons and produces behavioral changes such as high frequency of self‐grooming behaviors and tics (Aida et al., 2015). These abnormal behaviors are reduced with memantine treatment, an NMDA inhibitor (Aida et al., 2015), which suggests that hyper glutamatergic activity via NMDA is associated with this type of RRBs.

Neurotransmitters can also regulate the expression of some growth factors. The Epidermal Growth Factor (EGF) is involved in cell proliferation, survival, and differentiation (Reynolds et al., 1992; Reynolds & Weiss, 1996) via the epidermal growth factor receptor (EGFR) (Cohen et al., 1982). Toyoda et al. (2007) found a genetic association between EGF and ASD. Toddler and child patients with ASD (Onore et al., 2012) express low serum levels of EGF, which are inversely correlated to tip‐toeing behavior (Russo, 2013). In contrast, high EGFR expression positively correlates to sound sensitivity which suggests that the EGF/EGFR pathway may regulate these lower‐level RRBs in ASD patients (Russo, 2014). Transforming growth factor‐beta (TGF‐β1) is another growth factor that regulates proliferation, survival, and cellular fate. In patients with ASD, the expression level of TGF‐β1 during early childhood seems to be associated with stereotypy (Ashwood et al., 2008). In ASD brains, increased levels of TGF‐β1 were found in the MFG, anterior cingulate gyrus, and cerebellum (Vargas et al., 2005). However, in adult ASD patients, serum levels of TGF‐β1 also show a decrease that does not correlate to RRBs (Okada et al., 2007). Altogether, this evidence indicates that alterations in growth factors might be associated with lower‐level RRBs observed in ASD, but further experimental and clinical evidence is required to support this hypothesis.

ASD patients show abnormalities in brain size and cellular differentiation (Fang et al., 2014). In animal models for ASD, a polymorphism for the gen SERT (SLC6A4) induces an increase in the thickness of the cerebral cortex and hippocampus that correlates to self‐grooming and digging, which are analogous stereotypic behaviors in mice (Boylan et al., 2007). Interestingly, in toddlers with ASD, the same polymorphism has been strongly related to the elongation of gray matter in the frontal lobe (Wassink et al., 2007). However, it is unknown whether these morphological changes are related to lower‐level RRBs in people with ASD. Children, adolescents, and adults with ASD also show an increase in the volume of the right and left caudate nucleus which has a positive correlation with the manifestation of lower level of RRBs as complex mannerisms (Sears et al., 1999). In toddlers and early children with ASD present a reduced volume in the right globus pallidus and the left‐ and right‐side putamen. These reductions were associated with more severe stereotyped and repetitive behaviors (Estes et al., 2011).

A deficit in executive functions is involved in the development of lower‐level RRBs. ASD children with poorer control to respond proactively show more lower‐level RRBs (Faja & Nelson‐Darling, 2019). In adult patients, deficient motor interference performance predicts repetitive sensory‐motor behaviors (Mostert‐Kerckhoffs et al., 2015). Subjects with ASD manifest problems for proactively reducing the speed of motor responses that correlate with stereotypy (Schmitt et al., 2018). This evidence indicates that ASD courses with alterations in executive functions that modify improper behaviors and better adapt to a changing environment. These findings may help identify specific interventions that improve skills and the quality of life of people with ASD.

From the perspective of functional analysis it has been suggested that RRBs are shaped and maintained by themselves via self‐stimulation and perceptual reinforcement. In children with ASD, the blockage of sensory consequences of the stereotypy with gloves help reduce the hair‐manipulation behavior, a type of lower‐level RRBs (Rapp et al., 2000), whereas providing edible supplements instead of inedible items helps reduce the mouthing impulse (Lanovaz & Argumedes, 2010). In adolescents with ASD, atypical behaviors as hand waving, nose touching, body rocking, head movements, and object manipulation are not related to their reinforcements (Kennedy et al., 2000). Interestingly, the exposure to environmental enrichment before the stereotypy can decrease this type of lower‐level RRBs (Horner, 1980). Specifically, environmental changes are more effective in children and adults as compared to adolescents for reducing pica, self‐injurious, and motor stereotypy (Gover et al., 2019). Additional evidence indicates that a previously chosen and correctly executed behavior that is reinforced later help reduce lower‐level RRBs, specifically hand flapping, hand biting, screaming, pica, palm rubbing, playground scaping, hitting, kicking, ground lying, swinging, and rock lining (Machalicek et al., 2009). On the other hand, interruption of RRBs or child guiding to do other tasks, decreases body, hand (Gould et al., 2019), and vocal stereotypies (Pastrana et al., 2013). When inhibitory stimulus control is combined with response interruption and redirection, the behavioral benefit on lower‐level RRBs increases (Falligant & Dommestrup, 2020). Therefore, this strategy of matched stimuli may be functionally equivalent to the stimulation of repetitive behaviors (Lanovaz et al., 2010). Notably, RRBs appear to diminish aversive situations or experiences in people with ASD, for example, ear covering seems to reduce annoying noises (J. Tang et al., 2002). Hence, the stereotype is not maintained by itself but it seems to be reinforced and maintained by the consequences that follow these behaviors (Rapp & Vollmer, 2005).

## 5 BIOPSYCHOLOGICAL CORRELATES OF HIGHER‐LEVEL BEHAVIORS

Alterations in the dopaminergic system are common in higher‐level behaviors of RRBs. Specifically, a mutation neuroligin‐3 (NL3), a synaptic adhesion gene, was found in higher‐level behaviors. This evidence suggests that NL3 reduces the synaptic inhibition provided by DRD1 in neurons of the ventral striatum (Burrows et al., 2015). In children, adolescents, and adults with ASD, the gene that codifies the D3 receptor (DRD3) has been related to the behavior of insistence on sameness (Staal et al., 2012) and larger volumes of the striatum (Staal et al., 2015). Children with ASD also show an alteration in DRD4 that is associated with an increase in the frequency of obsessions and compulsions (Gadow et al., 2010). In contrast, adults with ASD also show low levels of the protein transporter for serotonin in the thalamus which seem to be associated with obsessive and repetitive behaviors in these patients (Nakamura et al., 2010), whereas children and adults with ASD show a disequilibrium in the expression of GABRB3 subunit (Cook et al., 1998). Remarkably, the severity in the insistence of sameness correlates to high expression levels of GABRB3 (Shao et al., 2003) and this reduction in the release of GABA into the striatum also has been related to compulsive behaviors (Chao et al., 2010). In this regard, a decrease in the expression of allopregnanolone, a neurosteroid agonist of GABA_A_ receptors, has been related to highly specific interests (higher‐level RRBs) (Chew et al., 2021).

The size of certain brain regions has also been linked to higher‐level RRBs, Hardan et al. (2006) found that children and adolescents with ASD present small sizes in the right lateral OFC, whereas adults with ASD have an increase in this region. Interestingly, the OFC correlates with the behavior of circumscribed interest, which supports the involvement of OFC in the incidence of higher‐level RRBs (Hardan et al., 2006). OCD shares some clinical characteristics with ASD, including compulsive behaviors and reduced gray matter in the medial OFC, anterior paracingulate cortex, and insula‐opercular (Pujol et al., 2004). All these regions have been related to severe obsession for checking behavior, whereas less gray matter in the right amygdala is also associated with higher‐level RRBs (Pujol et al., 2004). However, there is not enough evidence to link ASD with OCD.

Children, adolescents, and adults with ASD also show an increase in the volume of the right and left caudate nucleus, which has a positive correlation with the manifestation of higher‐level RRBs. A decrease in the volume of left cerebellar lobe VI and right cerebellar Crus I has a negative correlation with RRBs (Rojas et al., 2006). The enlargement in the right caudate has been associated with RRBs of higher level such as preoccupation, a circumscribed pattern of interest, and compulsive adherence to nonfunctional routines or rituals (Qiu et al., 2016). The increase in the caudate seems to persist until adolescence, where the enlargement of caudate (specifically in the right hemisphere) correlates with the insistence on sameness (Langen et al., 2014) and, in adults, it correlates negatively with compulsive behavior and difficulties for generating minor changes in routines (Sears et al., 1999).

Patients with ASD cannot control their attention and actions that, in turn, favors these patients become “*locked into*,” which is a line of thought or behavior that causes clinical manifestations such as echolalia, listening to a song repeatedly, strengths in one specific topic while suppressing others (Turner, 1997). Patients with ASD show deficient cognitive‐motor interference performance (Faja & Nelson‐Darling, 2019) and poor intentional motor inhibition, which may explain the impediment of these patients to halt properly a motor response (Schmitt et al., 2018).

Adolescent and adult patients usually commit mistakes in cognitive flexibility that do not correlate with their age or IQ (Ozonoff et al., 2004) but, in adolescents, this impairment correlates with mannerisms (South et al., 2012) whereas, in adults, it correlates with the need for routines, improper behavioral transitions, idiosyncratic speech (D'Cruz et al., 2013), and an increase in regressive mistakes (Miller et al., 2015). In summary, problems with cognitive flexibility are sustained throughout the lifespan of ASD patients. Notably, this executive function is exclusively related to high‐order RRBs, probably because they produce patterns of rigid behaviors and thoughts, whereas mistakes associated with deficits in cognitive flexibility can influence RRBs in adolescence.

At the behavioral level, in adults with ASD and mild mental retardation, the positive reinforcement as social attention seems to motivate the maintenance of perseverative speech (Rehfeldt & Chambers, 2003), supporting the idea that reinforcements per se seem to be important to develop higher‐level RRBs. During childhood, the reward response decreases some higher‐level RRBs such as the repetitive use of contextual words or sounds (Lanovaz et al., 2014), delayed echolalia, singing fragments of songs, and repeating fragments of movies (Taylor et al., 2005). In contrast, in adolescents and adults, the frequency of repeating the same word or phrase (Schauer, 2017) or talking about certain topics tends to reduce when an alternative behavior is rewarded (Rehfeldt & Chambers, 2003). These findings suggest that the reinforcement of alternative and appropriate behaviors may be successful for decreasing the frequency of higher‐level RRBs, specifically for those associated with language. Behavioral extinction is another strategy to reduce RRBs. With this approach, a reward is no longer given (or experienced) to a person that persists in improper conducts (Skinner, 1953). This type of learning helps reduce the severity of RRBs as observed in children with ASD who showed an improvement in food selectivity (Tarbox et al., 2010) by teaching functional communication and delaying disruptive and ritual behaviors such as screaming or crying (Rispoli et al., 2014), whereas the perseveration in behavior (word/phrase repetition) might be maintained for escaping from demands or unpleasant situations (negative reinforcement) (Durand & Crimmins, 1987).

## 6 CONCLUSIONS

**FIGURE 3:**
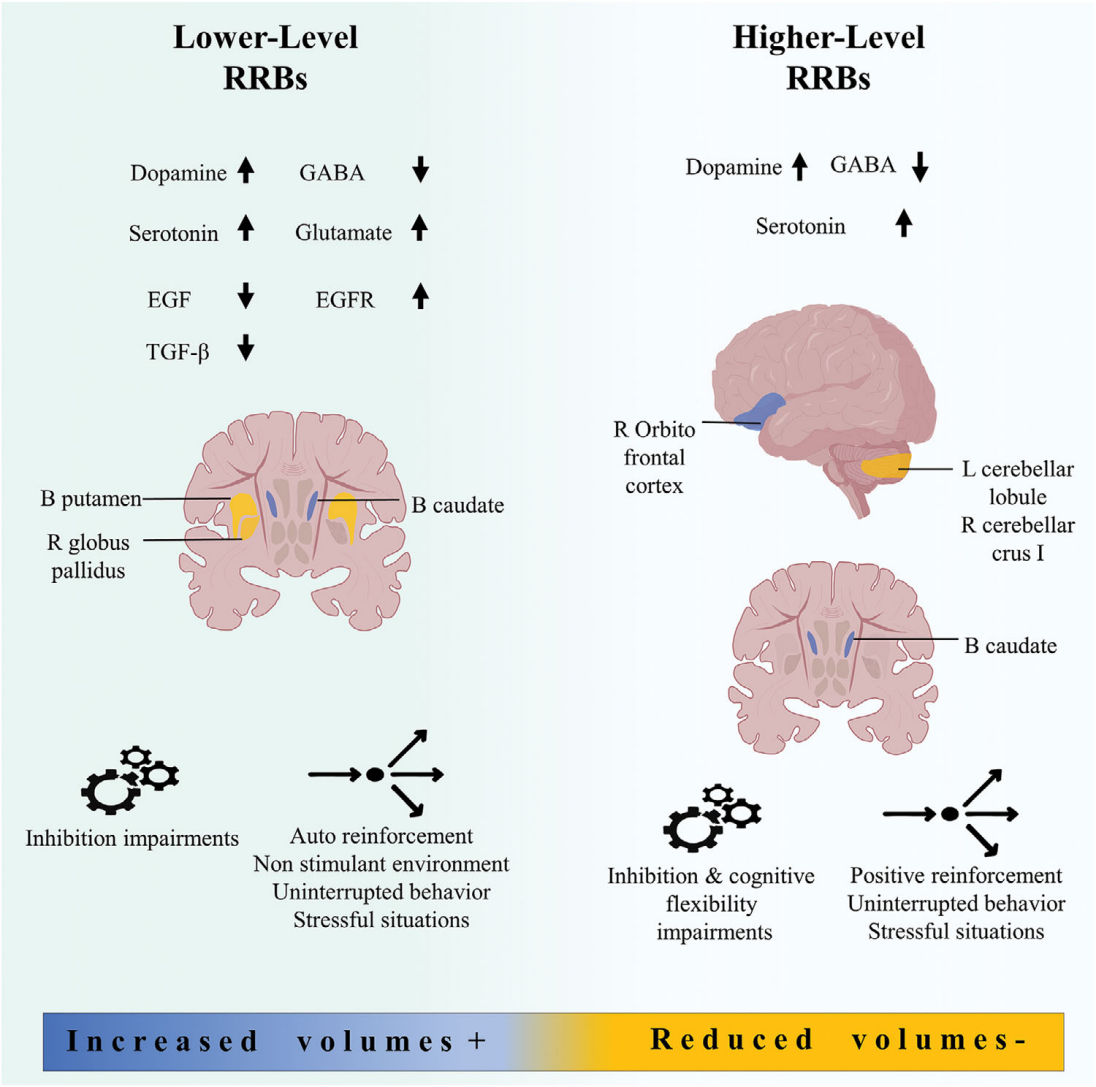
Schematic summary of the psychobiological correlates according to the type of RRBs. B, bilateral; L, left; R, right; **↑**, high levels of expression; **↓**, low levels of expression. Figure references: (Aida et al., 2015; Ashwood et al., 2008; Burrows et al., 2015; Chao et al., 2010; Ciaranello, 1982; Durand & Crimmins, 1987; Estes et al., 2011; Gould et al., 2019; Gover et al., 2019; Hardan et al., 2006; Lanovaz et al., 2010; Miller et al., 2015; Nakamura et al., 2010; Presti et al., 2003; Rehfeldt & Chambers, 2003; Rojas et al., 2006; Russo, 2013, 2014; Schauer, 2017; Schmitt et al., 2018; Sears et al., 1999; J. Tang et al., 2002)

RRBs comprise one of the two domains that characterize ASD but, unfortunately, these behaviors are not fully understood. Biopsychological correlates that have been involved in the origin of RRBs include: (a) A decrease in GABA associated with an increase in serotonin and glutamate, (b) alterations in the volume of the brain, (c) improper levels of growth factors, and (d) hypo‐ or hyperconnectivity among different brain regions. At the neuropsychological level, impairments in behavioral inhibition, cognitive flexibility, and monitoring for responses can make the integration of a dynamic environment difficult which, in turn, incites non‐adaptive behaviors. At the behavioral level, lower‐level RRBs seem to be maintained by providing stimulation, whereas non‐stimulating environments tend to increase these non‐adaptive behaviors. Nonetheless, the stimulation that RRBs provide does not explain the whole phenomenon, and the auto or hetero reinforcement of them seems to increase their frequency (Figure 3). Understanding the complexity of lower and higher level of RRBs could be useful to develop new behavioral and pharmacological strategies that help reduce the frequency and severity of this symptomatic domain that, in turn, improve the social and familiar adaptation of ASD patients.

## CONFLICT OF INTEREST

The authors declare no conflict of interest

### PEER REVIEW

The peer review history for this article is available at https://publons.com/publon/10.1002/brb3.2341

## Data Availability

Data sharing is not applicable to this article as no new data were created or analyzed in this study.
